# Patient Safety Culture in EU Legislation

**DOI:** 10.3390/healthcare8040410

**Published:** 2020-10-19

**Authors:** Anna Pilarska, Agnieszka Zimmermann, Kamila Piątkowska, Tomasz Jabłoński

**Affiliations:** 1 Department of Medical and Pharmacy Law, Medical University of Gdańsk, Tuwima 15, 80-210 Gdańsk, Poland; annapilarska@gumed.edu.pl (A.P.); kamila.piatkowska@gumed.edu.pl (K.P.); 2 Legal Department, European Medicines Agency, 1083 HS Amsterdam, The Netherlands; tomasz.jablonski@ema.europa.eu

**Keywords:** legislation, patient safety, patient safety culture

## Abstract

Patient safety means a condition in which a patient does not suffer any unnecessary actual harm, nor is exposed to any potential harm related to healthcare. The World Health Organization’s recognition of patient safety, as one of the most important factors in determining high quality healthcare, initiated the systematic introduction of changes in the approach to this issue, both globally and on the level of individual healthcare service providers. In order to enhance the quality and ensure the safety of healthcare services provided, national, European Union, and worldwide institutions focus on the introduction of a so-called patient safety culture. The creation of this safety culture would not be possible without the establishment of its legal framework. The purpose of this article is to shed light on the legislative achievements of the European Union within patient safety, taking into consideration acts that summarize the level of implementation of individual recommendations. This study can be useful both for those who focus their scientific interests on the subject of patient safety and those who need concise information on the legislative measures of the Community in this respect, as well as for medical personnel who want to become acquainted with this issue without reading comprehensive legal acts.

## 1. Introduction

The principle of primum non nocere has constituted a primary ethical rule in medicine since its very creation, but interest in developing a patient safety culture on a broader scale only started with two important reports published at the turn of the 21st century. The first was entitled, “To Err is Human” [1] and was published in 1999 by the *Institute of Medicine* in the United States as part of a larger project entitled “The Quality of Health Care in America”. The other report, entitled, “An Organization with a Memory”, was drawn up by the Chief Medical Officer of the United Kingdom in 2000 [2]. In both documents, the level of occurrence of adverse events during hospitalization oscillated around 10%, and, according to British scientists, only half of these adverse events were unavoidable, while the other half could have been prevented. Most adverse events were hospital-acquired infections, suffered by about 1 out of 20 hospitalized patients [2]. Data collected in “To Err is Human” also revealed that in the United States more people die annually due to medical mistakes than due to car accidents, breast cancer, and AIDS. Such research, while shocking to the general public, was not meant to be an attack against medical personnel or the healthcare system, but it was rather aimed at drawing attention to the fact that medical errors and their consequences constitute one of the most important problems in health protection. After all, the presented data showed that the most common cause of errors was not carelessness or lack of professionalism among medical personnel, but the improper organization of the healthcare system or the lack of an interdisciplinary approach to patient care. It was stressed that revolutionary changes in the healthcare system might not only introduce improvements, but also new hazards. The economic and social aspects of adverse events were analyzed as well as the problem of loss of trust to the healthcare system among citizens. It was proven that hospital-acquired infections caused extended hospitalizations, generated litigation costs, and led to loss of income, disabilities, and expenditure on additional treatments. The scale of such costs, in certain countries, is from a few to several dozen billion USD per year [1,2]. An essential element included in the American report was concrete solutions aimed at raising the level of patient safety. The proposals included in “To Err is Human” are an important point of reference to this day, both for global and national strategies that concern this issue. The most important solutions indicated in the publications included the need to create a national system of reporting adverse events, encouraging the practice of reporting such measures, and drawing conclusions therefrom. Moreover, the need to introduce tools of control and supervision over safety was noted and the necessity to withdraw from penalizing those who report adverse events was suggested. Otherwise, there would be a concern that the data supplied to the system would be incomplete, as medical personnel would not be willing to provide information. An important message of the report was also the suggestion that knowledge and experience within safety should be derived from other high-risk sectors [1]. Aviation is a very good example, where the results of human factor tests are used to enhance safety. It was recognized that if human error is something unavoidable in this sector, a system should be developed aimed firstly at minimizing the negative consequences of this, and secondly, giving the chance to draw valid conclusions based on errors already made, in order to minimize the risk of their recurrence [3].

The fact that the United States and the United Kingdom are not exceptional on a global scale, and patient safety is a worldwide problem, is confirmed by the data mentioned in “An Organization with a Memory”, and derived from similar research carried out in Australia, where the percentage of adverse events was even higher, in excess of 16%, and as in the United Kingdom, more than half of the instances were recognized as avoidable [2]. Similar data are presented in comparative analyses conducted on an international scale. Medical errors that are considered to be avoidable constitute 45.5% of cases, while other adverse events are classified as unavoidable. The accumulated probability of serious harm among patients with complications related to medical care amounts to 11.8%, and of unexpected death to 5.3%. Deaths caused by adverse events constitute 24.9% of causes of deaths in hospitals and 9.7% of deaths in total [4]. Research into the phenomenon of adverse events is also carried out in Member States of the European Union. According to the technical report Improving Patient Safety in the European Union, prepared for the European Commission in 2008, the percentage of adverse events in Member States was 8–12% of hospitalized patients. It is estimated that 4.1 million patients in the EU suffer from infections due to healthcare every year, and at least 37,000 people die for this reason [5]. Patients most often were exposed to infections, errors in drug administration, surgical errors, failures of medical equipment and diagnostic errors [6]. The core concern of this paper is to review legal regulations concerning adverse events, intentionally excluding those regulations concerning adverse drug events which are analyzed in the next part of the project.

Although the statistical data on adverse events have not presented a significant decrease in the last few years, there is an observed change in how the problem is perceived. From an issue that was initially of interest to just a few representatives of academic circles, it has now become a primary problem for most healthcare systems. The World Health Organization has recognized that safe healthcare is one of the most important factors determining high quality healthcare, and it requires global action in this respect [3]. Therefore, in response to World Health Assembly Resolution 55.18, the World Alliance for Patient Safety was founded in 2004. Since 2009, it has operated under the name WHO Patient Safety. As its major objective, the Alliance sets the coordination and acceleration of worldwide action for enhancing patient safety. This initiative aims to support the development of best practice policies within patient safety in all the member states of this organization. The actions of the Patients for Patient Safety programme are the priority of the initiative. The World Health Organization recognized that patients are the only entities that participate in the whole therapeutic process; therefore, their observations may be extremely valuable. Nevertheless, compared to doctors and other healthcare personnel, the recipients of healthcare services seldom have a chance to participate in projects that concern safety or in improving the quality of healthcare [3]. The World Health Organization, in order to take advantage of the experiences of patients, and to promote their leading role and involvement in actions for safety on every level of care, established the global Patients for Patient Safety network within the Patients for Patient Safety programme. The network is a group of people, including many patients, who have personally suffered harm during medical care. Due to what were often difficult experiences, they have become an important voice in the discussion on safety. They do not represent the World Health Organization nor speak on its behalf, but they speak for the policy of the World Health Organization in the improvement of healthcare quality and the safety of patients. It is also noteworthy that the patients themselves show a readiness to participate actively in improving the level of healthcare safety, and their involvement may be important on many levels, from direct contact with medical personnel in healthcare centers, through taking the opinions of patients into consideration when making changes in the management and organization, to planning the state health policy. In practice, the involvement of patients can be encountered most often in their direct contact with medical personnel [7]. Research shows that the involvement of patients on the hospital level may improve the safety of hospitalized patients in relation to medical personnel following hygiene standards, the prevention of errors in pharmacological therapy as well as the elimination of errors in surgical operations (for example, mistaking the side of the body) [8]. When analyzing the impact patients have on their own safety, it is also worth mentioning a very interesting experiment conducted in Israel which was to show what influence the behavior of patients and their families may have on the results of therapy achieved by medical teams. The simulation was carried out at neonatal intensive therapy wards, and their personnel were divided into teams. Some of the teams experienced negative comments and impolite or even rude behavior when fulfilling their duties, while other teams worked in neutral conditions. A comparison of the results of the work carried out by both groups revealed that when the attention of personnel was distracted by rudeness, this had a negative impact on the efficiency of diagnostics, the quality of work, team communication, and the flow of information among team members [9].

## 2. Materials and Methods

In this paper, the official database of European Union legal documents—Eur-Lex was used. In the database, the criterion of combining two words “patient safety” was applied. Then, the results were narrowed to legal instruments published in the Official Journal: directives, communications, recommendations—excluding legal acts concerned with the safety of medical products, medicines, food supplements, and disinfectants. The restriction was the result of the current stage of the project—in the following sections the safety of medicines, medial products, biocidal products, food supplements, and disinfectants are examined. In addition, the historical method presenting the changes in legislation was applied. The chronological outline of the implemented regulations was presented as well. Due to the fact, the criterion of the date of entry into force of legal acts was not treated as an element of inclusion or exclusion from the analysis.

## 3. Results

Taking into consideration the inclusion criteria we found: 1 declaration, 1 communication, 2 European Union Council conclusions, 2 recommendations and 3 reports. There are no hard law legal acts according to patient safety culture in European Union law system. As a result of the analysis the following soft law instruments were found. The Luxembourg Declaration on Patient Safety [10] from 2005 outlined the basic rules to be followed in national healthcare systems. In 2006, the current actions for the shared values and principles of the European Union concerning the method of how healthcare systems fulfill the demands of the population and patients were summarized in the Council Conclusions on Common values and principles in European Union Health Systems [11]. This declaration of 25 health ministers of the European Union concerns the common values and principles that underlie healthcare systems in Europe. In 2007, in the recommendation White Book entitled, “Together for Health: A Strategic Approach for the EU 2008–2013” [12], the European Commission formulated four principles that have underlain health strategy in the Community since 2013. In 2008 the “Communication from the Commission to the European Parliament and the Council on patient safety, including the prevention and control of healthcare-associated infections from 2008” [13] was officially published. In 2009 Council Recommendation of 9 June 2009 on patient safety, including the prevention and control of healthcare-associated infections (2009/C 151/01) [14] was introduced. The implementation of its content in Community countries was described in two reports created in 2012—European Parliament resolution of 22 October 2013 on the report from the Commission to the Council on the basis of Member States’ reports on the implementation of the Council Recommendation (2009/C 151/01) on patient safety, including the prevention and control of healthcare-associated infections (2013/2022(INI)) [15] and in 2014—The Commission’s Second Report to the Council on the Implementation of Council Recommendation 2009/C 151/01 on Patient Safety, Including the Prevention and Control of Healthcare Associated Infections [16]. In 2014 Patient Safety and Quality of Care Report was published by European Commission [17]. The European Union adopted in December 2014 Council Conclusions on Patient Safety and Quality of Care, Including the Prevention and Control of Healthcare-Associated Infections and Antimicrobial Resistance (2014/C 438/05) [18].

## 4. Discussion

The European Union has rich legislative achievements in the area of healthcare quality and patient safety, although the first such accomplishments were characterized by a high level of generality. The results of work conducted by high ranking officials in member state healthcare systems entitled “Patient Safety—Making it Happen” and the proposals included in this document were directed to institutions of the European Union, state authorities of Member States, service providers, and healthcare personnel. The post-conference recommendations for European Union institutions included, among others, a call to establish a Community forum to discuss European and national actions concerning patient safety as well as starting to cooperate with the World Health Organization Alliance in order to jointly understand the matter of patient safety and the establishment of the so-called “European Union solution bank” including examples of best practices and standards. A similar recommendation on discussing and taking measures within patient safety was submitted to the domestic authorities of Member States. In the recommendations for the institutions of the European Union, it was pointed out that initiatives in patient safety were to be supported by the Directorate-General for Health and Consumer Protection, renamed in 2015 the Directorate-General for Health and Food Safety. The competences of the Directorate-General include the protection of public health, ensuring the safety of food in Europe, caring for the health and conditions of animal breeding and the health of cultivated plants and forests. An important aspect that was referred to in the Declaration on Patient Safety [10] was the necessity to include the question of safety in regulations that concern drugs, medicinal products, and medical services, as well as a suggestion to draw up international standards on the safety and efficiency of medical technology. Moreover, attention was focused on the necessity to protect the confidentiality of the medical documentation of patients, with the simultaneous provision of access to necessary information for healthcare personnel. This suggestion is directed both towards European Union institutions and authorities on a national level. The latter group of recipients was dedicated the largest part of the Luxembourg Declaration [10]. First of all, the necessity to provide patients with full and free access to their health information was underlined. It is worth mentioning that this does not refer solely and exclusively to the accessibility of medical documentation, but also to informing a patient adequately and to how important it is for a patient to understand medical information received from medical personnel. The content of the Declaration [10] also includes the suggestion to take into account the benefits that stem from the operation of adverse event reporting systems and the introduction of risk management procedures as well as the use of new technologies. Other recommendations concern the issue of the sufficient preparation of medical personnel. One of the most important recommendations, which was subsequently referred to many a time in consecutive legal acts, both on the European Union and national levels, was the requirement to include the subject of patient safety in the curricula of healthcare employees and to enable medical personnel to attend training sessions provided by the manufacturers of medical equipment, thus giving them an opportunity to use such equipment safely and take full advantage of opportunities offered by new technologies. The issue of the working conditions of employees of the healthcare sector and their impact on patient safety was also raised. The last recommendation aimed at domestic authorities concerned the development of a patient safety culture of learning from errors and lessons learnt, without the need to focus on the punishment of liable persons [10]. Recommendations for healthcare entities and medical practitioners were limited to three issues, which concerned primarily the cooperation between them and taking patient safety into account in their collaboration. This part of the document also covered the important matter of cooperation between the patients and their relatives and medical practitioners, in order to deepen their awareness of medical events [10].

In Council Conclusions on Common values and principles in European Union Health Systems [11] the following values were listed as the most important and common for the whole of Europe: universality, access to good, quality care, equity, and solidarity. It was also explained how to understand the above-mentioned principles: “Universality means that no-one is barred access to healthcare; solidarity is closely linked to the financial arrangement of our national health systems and the need to ensure accessibility to all; equity relates to equal access according to need, regardless of ethnicity, gender, age, social status or ability to pay” [11]. It was also stressed that healthcare systems of Member States should aim to reduce the gap in health inequalities, including by the promotion of healthy lifestyles and other forms of illness prevention.

Making the healthcare systems financially sustainable is a guarantee of safeguarding the above-mentioned values. It was also indicated that the collection of principles that are common for the whole European Union is the foundation of the most important values, such as quality, safety, care based on evidence and ethics, patient involvement, redress, privacy, and confidentiality. One element that was missing in previous documents was that of paying attention to the need to base healthcare on ethical attitudes. This principle may seem obvious; however, in the explanation of this section, the question of technical advancement was included as well as demographic challenges when taking difficult ethical decisions. As an example, the following issue was mentioned, “the challenge of prioritizing healthcare in a way that balances the needs of individual patients with the financial resources available to treat the whole population” [11].

The first principle pointed in White Book entitled “Together for Health: A Strategic Approach for the EU 2008–2013” [12] is a strategy based on shared health values, such as citizen empowerment, rights of patients, scientific evidence, and reducing inequities in health. The second aspect, which was particularly focused on, concerned economic issues and recognizing the principle that health is the greatest wealth. According to this principle, all spending on health in the healthcare sector should be treated as an investment. It also focuses on the awareness that a lack of sufficient investment causes increased medical costs in the future. The third principle of the White Book is the inclusion of health issues in numerous policy areas by the Commission, not only in those that are directly related to health. The last principle of the health strategy is initiating cooperation with partners from outside the community, including the World Health Organization and other international organizations [12].

The document “Communication from the Commission to the European Parliament and the Council on patient safety, including the prevention and control of healthcare-associated infections from 2008” [13], apart from the general guidelines known already from previous legislative measures, focuses attention primarily on the problem of infections related to healthcare, which are among the most common causes of unintended harm. It is estimated that such infections effect on average one out of twenty hospitalized patients, which means 4.1 million patients in the European Union every year. Healthcare-associated infections are sometimes particularly difficult to cure, due to the resistance of the microorganisms that cause them to antibacterial measures. It has been shown that a number of factors affect this state of affairs, including organizational and behavioral factors. They include problems such as a high level of hospital bed occupancy, frequent movements of patients within healthcare systems and between them, an insufficient number of personnel compared to the number of patients, the failure to follow hand hygiene standards, and other rules of prophylactics and infection control as well as the improper use of medicinal products introduced into organs and body cavities by medical personnel. In the general assessment of patient safety, all measures aimed at improving the safety of drug use were evaluated positively, although actions for the enhancement of patient safety as a whole were perceived as insufficient to meet all the needs of patients. Such measures, according to the Commission, focus on individual causes or factors and do not concern general barriers that hinder the enhancement of safety and result from culture, management style, systems, communication, and process organization [13].

The most important and concrete legislative measure for the improvement of patient safety in the European Union to date was the Council Recommendation of 9 June 2009 on patient safety, including the prevention and control of healthcare-associated infections (2009/C 151/01) [14]. The document consists of two parts that include recommendations and a third part that obliges Member States to disseminate the content of recommendations in healthcare organizations, professional bodies, and educational institutions and report to the Commission on the progress of implementing the Council Recommendation. The first chapter, which concerns general patient safety issues, exhorts Member States to introduce a number of measures in order to minimize the harm suffered by patients when taking advantage of healthcare. Such measures include, among others, establishing institutions in every country responsible for patient safety, updating safety standards, and disseminating the best practices in healthcare. The content of the Recommendation also pays attention to the significant influence of information and communication technologies on improving patient safety. The examples given include electronic health records and e-prescriptions [14]. Such solutions are also an important factor in raising the quality and enhancing the accessibility of care. An example may be electronic systems of patient registration, which, on the one hand, save a patient’s time when calling a healthcare entity to make or cancel an appointment, and on the other hand, owing to reminding a patient automatically of an appointment, reduce the number of situations where a patient does not appear for an appointment without prior notice. However, what is most important is stressed in the first chapter, that patient safety has to become a primary subject both in national and local healthcare programs.

Since the publication of Recommendation 2009/C 151/01, the European Commission has drawn up two reports on the implementation of its content in Community countries. The first report was created in 2012 [15] and a consecutive one in 2014 [16], being to this day the most up-to-date collective document including data gathered in reports on a national level. The serious approach of European Union countries to the subject of patient safety may be proven by the fact that the report from 2012 already included information that “all states have developed policies in patient safety or have taken them into account as a priority issue in their healthcare policy. In 19 Member States, a competent authority responsible for patient safety on a national or regional level was officially established based on a legal act, and in six other countries it was appointed without a legal act” [15]. In most countries, patient safety has been recognized as a priority in the health policy of the state, and the authorities concerned have been given competences in implementing and supervising the implementation of the assumptions of the Recommendation [17]. On the other hand, in the Council Conclusions on patient safety and care quality, including the prophylactics of healthcare-related infections and the resistance to antibacterial measures, and the control of such phenomena (2014/C 438/05) [18], work carried out by the Working Group for Patient Safety and Care Quality on practical guidelines for education and training and the system of reporting adverse events and drawing relevant conclusions was evaluated positively. Nevertheless, the greatest concern is raised by the data referred to in the Conclusions [18] and published by the European Centre for Disease Prevention and Control (ECDC), on the growing number of cases of infections caused by drug-resistant microorganisms [18,19].

## 5. Conclusions

When analyzing the legislative achievements of the European Union within patient safety, one has to notice that on the community level no new regulations have come into effect for several years. This does not mean that the subject of patient safety has lost its importance in recent years. Any and all changes, in particular on an international level, need years before their effects are felt in practice. Legal acts to date include numerous aspects of the issue of patient safety, and require, first of all, a number of legislative measures on a domestic level. Moreover, for the further development of the level of patient safety and quality in healthcare, it is essential that legal solutions, worked out on both a community and domestic level, have an actual influence on actions on the healthcare center level, rather than operating only in theory. Therefore, concrete and binding regulations have to come into being on a domestic level; moreover, competences have to be granted to the relevant authorities, which will ensure compliance with such regulations.

Another stage of implementing changes in patient safety is the development of the conviction that they are needed and favorable, both for medical personnel and patients. At this stage, the relevant education of persons who practice medical professions should play a key role. Legislative changes, especially those that impose any additional duties, may raise doubts among medical staff. Such doubts may result from two very important factors. First of all, concerns that the growing interest in raising the patient safety level may increase the frequency and severity of any consequences which arise for those who have contributed to posing any risks for patients. Secondly, the present lack of education in patient safety may give the impression, especially among more experienced personnel, that the introduction of any changes to the daily work routine is unnecessary. In view of such concerns, one has to bear in mind that the introduction of any legislative changes to raise the quality of healthcare and patient safety has to be accompanied by suitable education for medical personnel. The proper education of personnel is also necessary to ensure patients are adequately informed and provide them with knowledge on how to raise their own level of safety in the course of their treatment, and also, indirectly, to build social awareness of a patient safety culture.
